# Incidence of cervical dysplasia and cervical cancer in women living with HIV in Denmark: comparison with the general population

**DOI:** 10.7448/IAS.17.4.19646

**Published:** 2014-11-02

**Authors:** Kristina Thorsteinsson, Steen Ladelund, Søren Jensen-Fangel, Terese Lea Katzenstein, Isik Somuncu Johansen, Gitte Pedersen, Jette Junge, Marie Helleberg, Merete Storgaard, Niels Obel, Anne-Mette Lebech

**Affiliations:** 1 Department of Infectious Diseases, Copenhagen University Hospital, Hvidovre, Denmark; 2 Clinical Research Center, Copenhagen University Hospital, Hvidovre, Denmark; 3 Department of Infectious Diseases, Aarhus University Hospital, Aarhus, Denmark; 4 Department of Infectious Diseases, The National University Hospital, Rigshospitalet, Copenhagen, Denmark; 5 Department of Infectious Diseases, Odense University Hospital, Odense, Denmark; 6 Department of Infectious Diseases, Aalborg University Hospital, Aalborg, Denmark; 7 Department of Pathology, Copenhagen University Hospital, Hvidovre, Denmark

## Abstract

**Introduction:**

Women living with HIV (WLWH) are reportedly at increased risk of invasive cervical cancer (ICC). WLWH in Denmark attend the National ICC screening program less often than women in the general population. We aimed to estimate the incidence of cervical dysplasia and ICC in WLWH in Denmark.

**Methods:**

We studied a nationwide cohort of WLWH and a cohort of age-matched females from the general population in the period 1999–2010. Pathology samples were obtained from The Danish Pathology Data Bank containing nationwide records of all pathology specimens. The cumulative incidence and hazard ratios (HRs) for time from inclusion to first cervical intraepithelial neoplasia (CIN)/ICC and time from first normal cervical cytology to first CIN/ICC were estimated. Sensitivity analyses were performed to include prior screening outcome, screening intensity and treatment of CIN/ICC in the interpretation of results.

**Results:**

We followed 1,143 WLWH and 17,145 controls with no prior history of ICC for 9,509 and 157,362 person-years. Compared to controls, the overall incidence of CIN1 or worse (CIN1+), CIN2+ and CIN3+ was higher in WLWH and predicted by young age and CD4 count <200 cells/µL. In women with normal baseline cytology, incidences of CIN1+ and CIN2+ were higher in WLWH. However, incidences were comparable between WLWH and controls adherent to the National ICC screening program.

**Conclusions:**

Overall, WLWH develop more cervical disease than controls. However, incidences of CIN are comparable amongst WLWH and controls adherent to the National ICC screening program and with a normal baseline cytology.

